# Physiological and Pathological Role of Circadian Hormones in Osteoarthritis: Dose-Dependent or Time-Dependent?

**DOI:** 10.3390/jcm8091415

**Published:** 2019-09-08

**Authors:** Farhad Md. Hossain, Yunkyung Hong, Yunho Jin, Jeonghyun Choi, Yonggeun Hong

**Affiliations:** 1 Department of Physical Therapy, Graduate School of Inje University, Gimhae 50834, Korea; 2 Ubiquitous Healthcare & Anti-Aging Research Center (u-HARC), Inje University, Gimhae 50834, Korea (Y.H.) (Y.J.) (J.C.); 3 Biohealth Products Research Center (BPRC), Inje University, Gimhae 50834, Korea; 4 Department of Rehabilitation Science, Graduate School of Inje University, Gimhae 50834, Korea; 5 Department of Physical Therapy, College of Healthcare Medical Science & Engineering, Inje University, Gimhae 50834, Korea; 6 Department of Medicine, Division of Hematology/Oncology, Harvard Medical School-Beth Israel Deaconess Medical Center, Boston, MA 02215, USA

**Keywords:** osteoarthritis, melatonin, thyroid-stimulating hormone, cortisol, circadian clock

## Abstract

Osteoarthritis (OA), the most common form of arthritis, may be triggered by improper secretion of circadian clock-regulated hormones, such as melatonin, thyroid-stimulating hormone (TSH), or cortisol. The imbalance of these hormones alters the expression of pro-inflammatory cytokines and cartilage degenerative enzymes in articular cartilage, resulting in cartilage erosion, synovial inflammation, and osteophyte formation, the major hallmarks of OA. In this review, we summarize the effects of circadian melatonin, TSH, and cortisol on OA, focusing on how different levels of these hormones affect OA pathogenesis and recovery with respect to the circadian clock. We also highlight the effects of melatonin, TSH, and cortisol at different concentrations both in vivo and in vitro, which may help to elucidate the relationship between circadian hormones and OA.

## 1. Introduction

Osteoarthritis (OA) is a chronic, degenerative joint disorder characterized by progressive erosion of cartilage loss of extracellular matrix (ECM) molecules, including type II collagen (Col2a1), proteoglycans, and tissue fluid; and hypertrophy of bone at the margins. Biochemical and morphological changes in the synovial membrane and joint capsule due to imbalances in anabolic and catabolic factors further exacerbate joint damage, resulting in pain and swelling of affected joints [1]. OA is the most common form of arthritis, affecting approximately 3.8% of the global population (250 million people) [2,3]. Women over the age of 60 are disproportionately affected (18%) compared to their male counterparts (10%) [4]. Major risk factors for OA include aging, injury, inflammation, obesity, and mechanical wear and tear [5]. 

Col2a1 and proteoglycans are the main components of articular cartilage. Under normal conditions, chondrocytes present in the articular cartilage work to maintain the balance between Col2a1 and proteoglycans [6,7]. During the early stages of OA, mechanical overload induces bone remodeling, as well as subchondral bone loss. Increased bone remodeling and load transmission contribute to altering the joint shape and progressive cartilage loss [8]. In the intermediate stages of disease progression, the production of matrix metalloproteinases (MMPs), as well as a disintegrin and metalloproteinase with thrombospondin motifs (ADAMTSs), are increased, which leads to cartilage degradation [9,10]. In later stages of OA, excessive production of inflammatory cytokines and cartilage degenerative enzymes further exacerbate fibrillations, leading to the development of fissures, cartilage loss, and osteophyte formation [9]. Over time, this progressive deterioration leads to the development of pain in the joint and surrounding tissues, and decreases mobility and quality of life [11,12]. Given the nature of disease progression, cartilage degradation, synovial inflammation, subchondral bone sclerosis, and osteophyte formation are regarded as the major hallmarks of OA [13,14].

Circadian clock-regulated hormones, including melatonin, thyroid-stimulating hormone (TSH), and cortisol are strongly associated with OA. Melatonin (N-acetyl-5-methoxytryptamine), a major neuroregulatory hormone, is primarily secreted from the pineal gland, and plays a fundamental role in circadian rhythmicity [15]. Melatonin levels exhibit strong circadian alterations with respect to time, with high levels secreted during nighttime hours, followed by lower expression during the day [16]. This hormone has multiple biological functions, including the regulation of circadian rhythms [17], anti-inflammatory and cytoprotective effects [18,19], inhibition of osteoclast activity [20,21], and the regulation of bone metabolism [22,23], as well as various antioxidative effects [24,25]. 

TSH is regarded as a pituitary hormone that stimulates the thyroid gland to produce thyroxine (T4), followed by triiodothyronine (T3) [26,27]. The hypothalamus, located in the base of the brain, produces thyrotropin-releasing hormone (TRH), which in turn stimulates the pituitary gland to produce TSH [28,29]. TSH and other thyroid hormones are directly responsible for the maturation of chondrocytes [27], with TSH serving as a negative regulator of bone remodeling, preventing bone loss and suppressing bone turnover [30,31]. Interestingly, a recent study has found that the chondrocyte clock is regulated by N-methyl-D-aspartate receptors (NMDARs) [32]. In particularly, the NMDAR subunit GluN2B is expected to alter the chondrocyte clock, resulting in OA pathogenesis [32].

Cortisol, which plays a pivotal physiological role in human physiology, including the control of stress and inflammation, is a major steroid hormone secreted from the adrenal gland [33]. The production rate of cortisol is similar in children and adolescents, and the total amount of cortisol produced in 24 h is around 9.5–9.9 mg/day or 5.7–7.4 mg/m^2^/day [34,35]. Cortisol secretion is tightly regulated by the suprachiasmatic nucleus (SCN), the central clock of the hypothalamus [33]. The prime function of cortisol is considered to be as a secondary messenger between the central and peripheral clocks, which act to synchronize the body’s circadian rhythm. However, elevated levels of cortisol, mainly in the early morning session, aggravates stress, which results in activation of inflammatory cytokines [33,36]. 

The aim of this review is to elucidate the effects of circadian hormones (melatonin, TSH, and cortisol) based on their secretion patterns at different time points during OA progression. Furthermore, we also deal with variations between in vitro and in vivo OA models at different concentrations of melatonin.

## 2. Source and Synthesis of Circadian Hormones 

**Figure 1:**
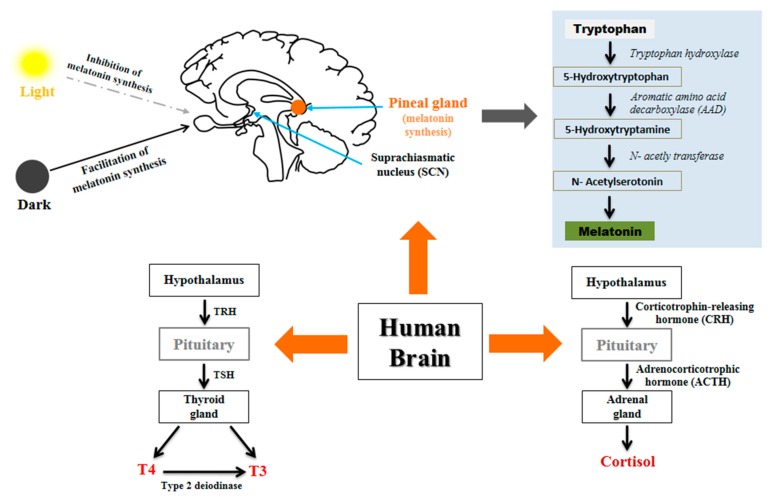
The synthesis of melatonin, thyroid-stimulating hormone (TSH), and cortisol, depending on circadian rhythm. The expression of cortisol, a steroid hormone produced in the adrenal gland, is tightly regulated by circadian rhythms in various mammals, including humans. The primary rhythm of this cycle is controlled by the suprachiasmatic nucleus (SCN), located in the hypothalamus. The secretion pattern of cortisol is coordinated by the hypothalamic–pituitary–adrenal (HPA) axis and the hippocampus. This HPA axis receives input from the SCN, from which it controls corticotrophin-releasing hormone (CRH) release in the paraventricular nucleus. From there, adrenocorticotrophic hormone (ACTH) is released from the corticotropes in the anterior pituitary by stimulating CRH. In normal individuals, cortisol levels fall to low or even undetectable levels around midnight, followed by peak expression around at 08:30.

Melatonin, a circadian clock-regulated hormone, is primarily secreted by the pineal gland of the brain, with lower levels of production from other organs, such as the retina, bone marrow, Harderian gland, pancreas, and kidneys [37]. Structurally, melatonin was first identified in 1958 [38], and has been described in a wide range of non-mammalian species, including plants, fish, and birds [39]. For melatonin synthesis, the retina absorbs light, which in turn produces a signal in the hypothalamus. This signal then moves to the paraventricular nuclei, followed by the superior cervical ganglion (SCG). Activation of the SCG enables the signal to pass through to the pineal gland, where, in the presence of norepinephrine, melatonin production follows [40,41]. From a biochemical standpoint, melatonin synthesis is best summarized as a three-step process, consisting of hydroxylation, decarboxylation, and acetylation (Figure 1). Primarily, tryptophan is hydroxylated in the presence of tryptophan hydroxylase to produce 5-hydroxytryptophan (5-HTP). Subsequently, 5-HTP is decarboxylated into serotonin by aromatic amino acid decarboxylase (AAD), after which it is acetylated by arylalkylamine N-acetyltransferase (AANAT) to form N-acetylserotonin, followed by conversion into melatonin via the action of hydroxyindole-O-methyltransferase (HIOMT) [37,40].

TSH, also known as thyrotropin, is a pituitary hormone first identified by Allen and Smith in 1916 [42]. TSH stimulates T4, which is converted into T3 via a type 2 deiodinase in tanycytes, specialized glial cells located in the third ventricle [43,44]. In humans, almost 100% of T4 is secreted from the thyroid gland, although only 20% of T3 is derived from this source, with the remaining 80% produced by the peripheral conversion of T4 to T3. The daily production rate of T4 (110 nM) is approximately double that of T3 (50 nM), and circulating levels of T4 are considered to be 3–4-fold higher than those of T3. Additionally, the half-life of circulating T3 is 0.75 days compared with 6.7 days for T4 [45]. TSH maintains a distinct circadian rhythm, with levels typically peaking between 02:00 and 04:00 and falling to their nadir from 16:00 to 20:00 [46,47]. Other hormones essential for the stimulation of thyrotropin-releasing hormone (TRH) to produce TSH include leptin and dopamine. Dopamine stimulates TRH through cognate neurons in the paraventricular nucleus [48], whereas leptin directly acts on the thyrotropic region of the paraventricular nucleus, resulting in stimulation of corticotrophin-releasing hormone (CRH) [43].

In contrast, three inter-communicating regions, including the hypothalamus, pituitary gland, and adrenal gland control the secretion of cortisol, and these regions are known as the hypothalamic–pituitary–adrenal (HPA) axis. The HPA axis accepts signals from the SCN, which stimulates the release of CRH. CRH then causes the pituitary gland to secrete adrenocorticotrophic hormones (ACTH) into the bloodstream. Finally, elevated levels of ACTH are detected in the adrenal glands, which significantly stimulate cortisol secretion [49]. Due to an increased level of cortisol, secretion of CRH and ACTH by the hypothalamus and pituitary, respectively, are blocked. As a result, ACTH levels fall, which then results in reduced cortisol levels.

## 3. Secretion Pattern of Melatonin

Melatonin plays a crucial role as a regulator of endocrine rhythms, as well as daily biorhythms [50]. In healthy people, melatonin secretion begins to rise early in the evening, and reaches maximum levels late at night, followed by progressive decreases thereafter. Melatonin is a light-sensitive hormone, with daytime levels decreasing to 0–20 pg/mL, compared with peak levels of approximately 60–200 pg/mL between early morning 02:00 and 03:00 [51]. The timing and magnitude of these rhythmic fluctuations in melatonin levels play a crucial role in regulating its various pro- and antioxidative effects [52], although its definitive effects remain unclear, as investigations into these effects are dependent on methodology, with clear differences observed between in vitro [53,54,55] and in vivo methods [56,57]. For example, the interrelationship between melatonin and oxidative stress was investigated by measuring vitamin E consumption in human red blood cells [53]. In this way, another study suggested that melatonin may modulate cellular redox status; however, whether this hormone plays a role as an intracellular antioxidant or not is unclear [55]. In rats exposed to oxidative damage, melatonin treatment was proved to reduce lipid peroxidation marker levels in the lung, liver, and serum of rat models [57].

### 3.1. Concentration-Based In Vitro Studies of Melatonin in Osteoarthritis

One of the most important functions of melatonin is its ability to serve as a scavenger of free radicals [58,59,60]; however, several in vitro studies have reported that high concentrations of melatonin promote reactive oxygen species (ROS) generation [55,61], and that these effects are dependent on the duration of melatonin treatment [52]. In addition, melatonin promotes oxidative activity in Jurkat cells, resulting in fas-induced cell death, with higher doses inducing significantly more ROS generation compared to low-dose treatments [55]. In these studies, ROS generation was increased from micromolar (µM) to millimolar (mM) levels, resulting in enhanced cell damage and the induction of apoptosis in resting primary neuronal cultures [62]. Similarly, an increase in oxidative stress marker expression was observed at a dose of 1 mM in an in vitro model of Alzheimer’s disease, using tissue culture sections; a reduction in oxidative damage was observed at <100 µM [63]. Interestingly, in HepG2 cells, lower concentrations of melatonin (0.1–10 µM) showed antioxidative effects at 24 h; however, by 96 h, these effects had become more pro-oxidant. These results suggest that the dynamism of glutathione was enriched within 24 h but reduced thereafter [61], suggesting that both the concentration and duration of melatonin treatment may affect its response to oxidative stress. These data suggest that high doses of melatonin promote ROS generation in vitro. Further support for such effects was reported by Hong et al. [64], who showed that concentrations of melatonin as low as 1 nM restored Col2a1, the main component of articular cartilage, via inhibition of active MMP-13. High concentrations of melatonin (1 mM) were unable to rescue the expression of Col2a1 after TNF-α exposure. The authors also suggest that high levels of melatonin may be responsible for the cytotoxic effects on TNF-α-induced chondrocytes (Table 1). 

**Figure 2:**
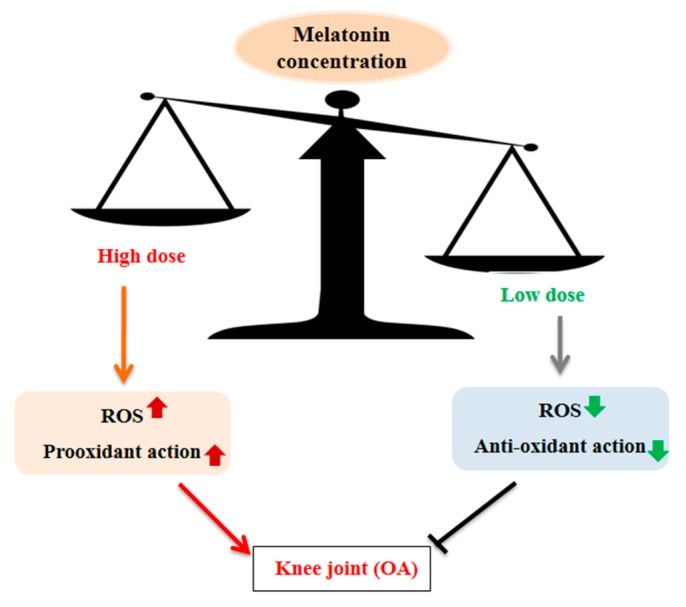
In vitro studies show that high concentrations of melatonin lead to increases in reactive oxygen species (ROS) expression, oxidative stress, and inflammatory cytokines, with low concentrations of melatonin exhibiting the opposite effects, including enhanced antioxidant action and reduced ROS expression. Low concentrations may inhibit cartilage degradation by regulating pro-inflammatory cytokines and ROS.

These studies revealed that high concentrations of melatonin increase ROS generation, which may promote the expression of pro-inflammatory cytokines and cartilage degenerative enzymes during OA progression. Furthermore, low concentrations of melatonin may restore the cartilage matrix through the inhibition of MMPs and ADAMTSs (Figure 2).

### 3.2. Concentration-Based In Vivo Studies of Melatonin in Osteoarthritis

Melatonin reduces oxidative stress via the induction of antioxidative enzymes [72,73,74]. Ozturk et al. [75] found that melatonin administration at a dose of 10 mg/kg increased superoxide dismutase (SOD) activity in rat liver. In addition, exogenous administration of melatonin (500 µg/kg) enhanced mRNA expression, not only of copper–zinc superoxide dismutase (CuZn-SOD), but also of manganese superoxide dismutase (Mn-SOD) in female Syrian hamsters [76]. Furthermore, melatonin injection (5 mg/kg) was shown to enhance SOD activity in the kidney, liver, and brain tissues of rats [77]. Treatment with either beta-amyloid peptide 25–35 [78] or D-galactose [79] induced oxidative damage in the brains of rats and mice; treatment with melatonin (0.1 to 10 mg/kg) restored SOD and glutathione peroxidase (GPx) activities. Similarly, melatonin (10 mg/kg) was also shown to protect against oxidative mitochondrial damage by increasing ATP production in the fetal brain, as well as stimulating GPx activity in the rat brain (Table 2) [80]. Because the administration of exogenous melatonin also increased the total antioxidant status (TAS) in rat serum, melatonin may be important in regulating the antioxidative capacity of rat serum [81]. Taken together, these studies indicate that administration of exogenous melatonin can promote antioxidative effects in various rodent models. 

**Figure 3:**
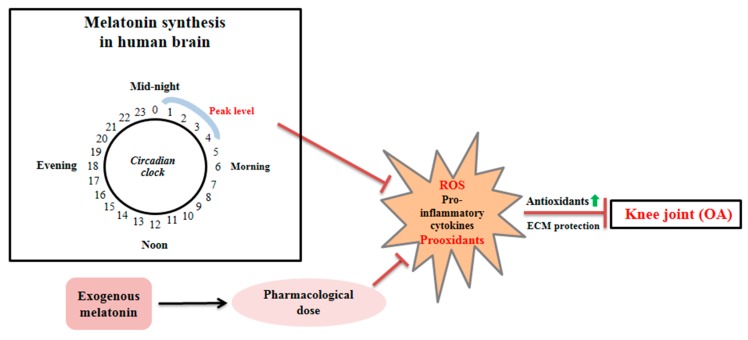
Endogenous melatonin suppresses excessive reactive oxygen species (ROS) production and pro-inflammatory cytokines, and increases antioxidant action during osteoarthritis (OA). In addition, pharmacological treatment with melatonin also shows beneficial effects that protect extracellular matrix (ECM) molecules in articular cartilage.

Endogenous melatonin concentrations are also regulated by the circadian clock, with plasma melatonin levels highest at midnight in both rats [81] and mice [85]. In another study, melatonin levels fell to their lowest point between 12:00 and 18:00, followed by a sharp peak thereafter, typically between 23:00 and 02:00 [86]. Pablos et al. [87] demonstrated that plasma melatonin levels are correlated with the total antioxidative capacity of the serum. Melatonin stimulates several antioxidative enzymes, including GPx. This enzyme exaggerates circadian rhythms, which are involved in the melatonin cycle. The authors found that SOD and melatonin exhibited vigorous circadian rhythms, with substantial overlap in the periodicity of these compounds. This suggests that the physiological enhancement of melatonin at night is directly related to the nocturnal increase in SOD expression. Further studies found that melatonin levels were suppressed in animals maintained under constant light exposure for seven days. SOD activity was also decreased in these animals, but alterations in the light/dark cycle elevated SOD activity. These results suggest that melatonin serves not only as a direct scavenger of free radicals, but also stimulates SOD activity, consistent with studies showing a strong correlation between melatonin concentration and antioxidant activity. Although the expression of antioxidative enzymes is suppressed due to excessive ROS generation during OA progression [88], there is no evidence that changes in melatonin concentrations affect ROS generation in vivo [56]. Together, these studies suggest that normal timing or high concentrations of melatonin may be beneficial for the inhibition of cartilage degeneration during OA progression (Figure 3).

## 4. Regulatory Effects of Melatonin on Osteoarthritis 

During OA progression, the prime component of articular cartilage, Col2a1, is broken down due to increased expression of pro-inflammatory cytokines, including IL-1, IL-6, and TNF-α, and tissue destructive enzymes such as MMPs and ADAMTSs [10]. MMP-13 is the most important enzyme responsible for cartilage destruction during OA [89]. Pineal gland-mediated production of melatonin has the ability to detoxify ROS or reactive nitrogen species (RNS) through its free radical scavenging capacity. Exposure to melatonin has been shown to increase the expression of chondrogenic marker genes, including Col2a1 and SOX-9, which promote matrix synthesis in articular chondrocytes and downregulate hypertrophic markers, such as collagen X [90]. Furthermore, melatonin treatment significantly reduces MMP-13 by inhibiting the phosphorylation of p38, ERK, JNK, and MAPK, and the activation of NF-κB [91]. Pro-inflammatory cytokines and other proteins, including IL-6, ADAMTS-4, and MMPs enhance catabolic processes, resulting in the destruction of the ECM, which in turn decreases the expression of anabolic pathways by inhibiting SOX-9 and Col2a1 [92,93]. However, melatonin has also been shown to inhibit IL-1β, IL-6, and TNF-α activity in mesenchymal stem cells via the induction of SOD activity [88,94]. Interestingly, Hong et al. [64] showed that melatonin treatment led to a decrease in MMP-13 expression in a collagenase-induced OA model, with melatonin intervention yielding better reductions in disease activity than melatonin alone. Furthermore, Rong et al. [95] found that osteoarthritic chondrocytes alter the expression of intrinsic circadian clock genes: brain muscle ARNT-like 1 (BMAL1) and period circadian regulator 2 (PER2). The peak level of PER2 was higher and the peak expression of BMAL1 was lower in damaged chondrocytes of an OA model compared with the control group. Concomitantly, knockdown of PER2 in the OA model attenuated the expression of cartilage-degenerative main enzymes, such as MMP-13 and ADAMTS-5. Interestingly, there was no significant change in circadian locomotor output cycles kaput (CLOCK), cryptochrome 1 (CRY1), cryptochrome 2 (CRY2), or period circadian regulator 1 (PER1) in damaged and undamaged chondrocytes. These results suggest that elevated expression of PER2 is responsible for OA development [95]. In addition, in vivo and in vitro studies demonstrated that decreased levels of BMAL1 is associated with OA pathogenesis [96,97]. It is interesting to note that melatonin alone or melatonin combined with exercise increased as well as restored BMAL1 expression in the collagenase-induced OA rat model [98]. Together, these data suggest that cartilage destruction may be reversed via the regulatory effects of melatonin, via the inhibition of pro-inflammatory cytokines, ROS production, and activation of chondrogenic marker genes, including Col2a1 and SOX-9.

## 5. Anabolic and Catabolic Effects of Thyroid-Stimulating Hormone on Articular Cartilage and Bone

**Figure 4:**
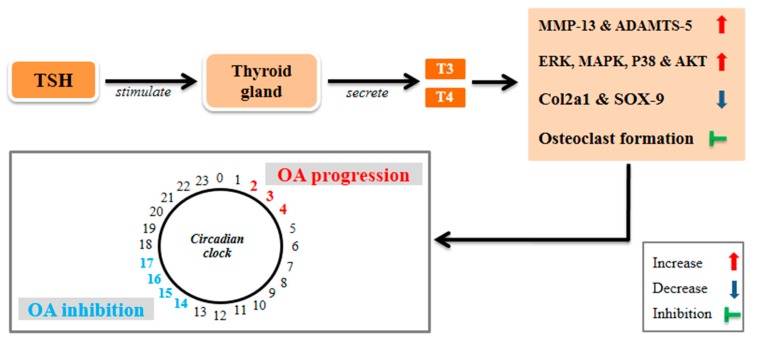
TSH stimulates the thyroid gland to secrete T3 and T4. TSH decreases Col2a1 and SOX-9 expression through activation of ERK, MAPK, and P38, as well as enhancing the expression of matrix metalloproteinase (MMP)-13 and a disintegrin and metalloproteinase with thrombospondin motif (ADAMTS)-5, which are responsible for cartilage degradation. The circadian clock-controlled TSH hormone shows peak levels shortly after midnight (02:00–04:00), which may promote OA. In contrast, lower levels, typically seen between 14:00 and 17:00, may inhibit OA progression.

Active thyroid hormone T3 stimulates collagen X matrix synthesis and the expression of alkaline phosphatase, and facilitates the progression of MMP-13, ultimately resulting in cartilage mineralization and degradation [99,100,101]. Furthermore, T3 enhances ADAMTS-5 and MMP-13 [102,103], which together promote the degradation of proteoglycans and Col2a1 [104,105]. Osteocalcin expression in MC3T3 cells is stimulated by T3 via activation of the AMPK pathway [106,107]. Similarly, TSH increases cAMP activity and reduces the levels of SOX-9 and Col2a1 in primary chondrocytes [108]. TSH activates ERK, MAPK, P38, and Akt signaling pathways in human osteoblastic U2OS-TSHR cells that overexpress TSH receptors [109]. Moreover, TSH significantly attenuates TNF-α gene expression and osteoclastogenesis in RAW-C3 cells and CD11b^+^ bone marrow cells [110,111]. Proinflammatory cytokines, including TNF-α, IL-1, and IL-6 stimulate osteoclast formation and activation, which increases bone loss, as well as osteoporosis and bone loss [110,112]. Interestingly, TSH exhibits a potent antiresorptive effect on bone and regulates osteoclast differentiation by suppressing TNF-α production [113]. Another study reveals that TSH inhibits osteoclast formation in RANKL-induced monocytic cell lines by inhibiting the JNK/c-jun and NF-κB signaling pathways [30]. In addition, TSH increases osteoblast differentiation, and low levels of systemically administered TSH enhances not only trabecular bone volume but also improves the bone’s mechanical strength in an ovariectomy rat model. These data indicate that TSH exerts both anabolic and antiresorptive effects on bone remodeling [31]. These results appear to demonstrate that overexpression of thyroid hormones initiate cartilage degenerative enzymes that are responsible for OA progression, and that systemic administration of low levels of TSH has positive effects on osteoblasts and bone remodeling. However, despite these observations, the rhythmic pattern of TSH secretion remains heavily dependent on circadian periodicity, which is controlled by endogenous oscillators and environmental synchronizers [114]. Russel et al. [45] showed that in healthy individuals, the secretion pattern of TSH reaches a peak level between 02:00 and 04:00 and a nadir between 16:00 and 20:00. This suggests that low-level secretion of TSH, such as that seen from 16:00 to 20:00, may offer a beneficial effect for the recovery of cartilage degeneration during OA progression (Figure 4).

## 6. Inflammatory Effects of Cortisol in Osteoarthritis

The steroid hormone cortisol is secreted from the adrenal gland, yet its effects remain tightly controlled by the body’s circadian rhythms. These rhythms are controlled by the SCN, located in the hypothalamus. The enzyme 11β-hydroxysteroid dehydrogenase (11βHSD1) actives glucocorticoids, and the expression of this enzyme significantly increases in osteoblasts with aging in humans and rodents [115]. Endogenous glucocorticoid metabolism in osteoblasts and osteocytes contributes to modulate the progression of OA [116]. Recently, Tu et al. [117] revealed that disrupted signaling of endogenous glucocorticoids attenuates cartilage damage, bone sclerosis, and osteophyte formation, which alleviates OA pathology. As with other circadian hormones, cortisol levels fall to very low or undetectable values around midnight, after which levels begin to increase again, starting in the early morning between 02:00 and 03:00, and peaking around 08:30, followed by a gradual decline thereafter (Table 3). 

Peak levels of cortisol are around 399 nM, compared to nighttime levels, which typically dip below 50 nM [118,119]. High levels of cortisol are associated with stress and lead to increases in NF-κB, IL-6, and other inflammatory mediators [36,120]. During OA, the expression of inflammatory cytokines, including IL-1, IL-6, and TNF-α, is increased, resulting in enhanced inflammation in chondrocytes [10]. In contrast, low concentrations of cortisol may decrease the likelihood of OA by helping to better regulate stress as well as inflammation.

## 7. Conclusions

Circadian rhythm-regulated hormones have a significant impact on OA pathogenesis by regulating the production of pro-inflammatory cytokines, cartilage degenerative enzymes, and inflammatory mediators. In this review, we summarized the role of circadian hormones, including melatonin, TSH, and cortisol, along with their various concentrations, in OA. Several in vitro studies have consistently shown that higher concentrations of melatonin lead to greater expression of pro-inflammatory cytokines, ROS, and cartilage-reducing enzymes, whereas lower concentrations inhibit OA progression. In contrast, high concentrations of melatonin not only promote its antioxidant effects, but also suppress ROS generation. Together, these results suggest that melatonin treatment, along with its different concentrations, may represent an important prognostic indicator for OA treatment. In addition, endogenous peak secretion of TSH and cortisol enhances cartilage degenerative enzymes, which leads to OA pathogenesis. As a result, lower levels of TSH and cortisol may contribute to lower OA severity. 

## Figures and Tables

**Table 1:** Effects of various concentrations of melatonin (in vitro).

Cell Line and Species	Melatonin Concentration	Effect of Melatonin with Dose Variation	Ref.
Primary cultured chondrocyte (rat)	10^−3^ M, 10^−6^ M and 10^−9^ M	10^−3^ M: Increased cytotoxic effect; high concentration failed to recover Col2a1 10^−6^–10^−9^ M: Inhibition of cell death, recovered cell surface area, and increased Col2a1 expression via MMP-13 inhibition.	[64]
HepG2 (human)	10^−3^–10^−4^ M 10^−6^–10^−8^ M	10^−3^–10^−4^ M: Increased pro-oxidant activity, increased ROS level after 96 h 10^−6^–10^−8^ M: Decreased cell viability, showed antioxidant action at 24 h	[61]
Jurkat T cell, (human)	(0.1–1) × 10^−3^ M	Increased ROS, fas-induced apoptosis occurred by decreasing antioxidant activity	[55]
MOLT-4, CMK, (human)	10^−3^ M	Increased cytotoxicity and ROS production	[65]
B6D2F1 (mouse)	10^−9^ and 10^−6^ M	10^-6^ M: increased ROS level and GSH level decreased compared with 10^−9^ M in oocytes.	[66]
U937 (human)	10^−3^ M	Increased ROS production and ameliorated GSH level	[67]
U937 (human)	10^−3^ M	NF-κB activation, ROS generation and apoptosis	[68]
Mouse 2-cell embryo (mouse)	10^−9^ M, 10^−3^ M	10^−3^ M: Possibility of cell injury and lower rate of blastocyst 10^−9^ M: Improved at maximum blastocyst rate and hatching blastocyst rate	[69]
HT22 and BV2 (mouse)	100 × 10^−6^ M	Reduced the elevated ROS and oxidative stress, reduced p38 MAPK Prevent apoptosis through the suppression of activated caspase-3	[70]
A-431, CCD- 1079Sk (human)	(0.03–0.125) × 10^−3^ M (0.125–5) × 10^−3^ M	(0.03–0.125) × 10^−3^ M: Increased cell proliferation, decreased ROS production (0.125–5) × 10^−3^ M: Leads to increase ROS, DNA damage, apoptosis, and decreased cell viability	[71]

**Table 2:** Effects of various doses of melatonin (in vivo).

Route of Administration and Animals	Dose of Melatonin	Effects	Ref.
Subcutaneous injection (rat)	10 mg/kg	Increased Col2a1 level through MMP-13 inhibition, suppressed pro-inflammatory cytokines, and catalytic transcription factors were found in OA knee.	[64]
Oral administration (mouse)	10 mg/kg	Prevented cytotoxicity, and increased serum SOD and glutathione (GSH) levels.	[82]
Intraperitoneal injection (rat)	20 mg/kg	Decreased apoptosis, repressed IL-1β and TNF-α in the spinal dorsal horn; anti-nociceptive effect.	[83]
Intraperitoneal injection (rat)	20 mg/kg	Reduced ROS and oxidative stress, activated antioxidant mechanism, and inhibited neuroinflammation by reducing NF-κB in mouse embryos.	[69]
Intravenous injection (mouse)	5, 10, or 20 mg/kg	Anti-inflammatory action through activating PPAR-γ; inhibited TNF-α, IL-1, and IL-6 production, and 20 mg/kg was more effective for reduction.	[84]
Subcutaneous injection (rat)	10 mg/kg	Increased SOD activity, decreased nitrite levels in the liver.	[75]
Subcutaneous injection (Syrian hamster)	500 µg/kg	Decreased percent of damaged cells, increased CuZn-SOD and Mn-SOD in the Harderian gland.	[76]
Intraperitoneal injection (rat)	5 mg/kg	Increased SOD activity and glutathione reductase in kidney, liver, and brain tissue.	[77]
Intragastric administration (mouse)	0.1, 1, or 10 mg/kg	Ameliorated SOD and CuZn-SOD in brain tissue.	[79]
Intraperitoneal injection (rat)	10 mg/kg	Prevented oxidative mitochondrial damage by the activation of glutathione peroxidase (GSH-Px) in brain tissue.	[80]

**Table 3:** Secretion pattern based on the circadian clock.

Circadian Hormones	Low Level	Mid-Level	Peak Level	Ref.
Melatonin	12:00–20:00	22:00–23:00	02:00–04:00	[48,80]
TSH	14:00–16:00	21:00–23:00	02:00–04:00	[44]
Cortisol	01:00–03:00	13:00–17:00	08:00–08:30	[114,118]

## References

[1] Kuettner K.E., Cole A.A. (2005). Cartilage degeneration in different human joints. Osteoarthr. Cartil..

[2] March L., Smith E.U., Hoy D.G., Cross M.J., Sanchez-Riera L., Blyth F., Buchbinder R., Vos T., Woolf A.D. (2014). Burden of disability due to musculoskeletal (MSK) disorders. Best Pract. Res. Clin. Rheumatol..

[3] Lim S.S., Vos T., Flaxman A.D., Danaei G., Shibuya K., Adair-Rohani H., Amann M., Anderson H.R., Andrews K.G., Aryee M. (2012). A comparative risk assessment of burden of disease and injury attributable to 67 risk factors and risk factor clusters in 21 regions, 1990–2010: A systematic analysis for the Global Burden of Disease Study 2010. Lancet.

[4] Cross M., Smith E., Hoy D., Nolte S., Ackerman I., Fransen M., Bridgett L., Williams S., Guillemin F., Hill C.L. (2014). The global burden of hip and knee osteoarthritis: Estimates from the Global Burden of Disease 2010 study. Ann. Rheum. Dis..

[5] Arden N., Nevitt M. (2006). Osteoarthritis: Epidemiology. Best Pract. Res. Clin. Rheumatol..

[6] Nakata K., Ono K., Miyazaki J., Olsen B.R., Muragaki Y., Adachi E., Yamamura K., Kimura T. (1993). Osteoarthritis associated with mild chondrodysplasia in transgenic mice expressing alpha 1(IX) collagen chains with a central deletion. Proc. Natl. Acad. Sci. USA.

[7] Sandell L.J., Aigner T. (2001). Articular cartilage and changes in arthritis. An introduction: Cell biology of osteoarthritis. Arthritis Res..

[8] Yuan X.L., Meng H.Y., Wang Y.C., Peng J., Guo Q.Y., Wang A.Y., Lu S.B. (2014). Bone–cartilage interface crosstalk in osteoarthritis: Potential pathways and future therapeutic strategies. Osteoarthr. Cartil..

[9] Almonte-Becerril M., Navarro-Garcia F., Gonzalez-Robles A., Vega-Lopez M.A., Lavalle C., Kouri J.B. (2010). Cell death of chondrocytes is a combination between apoptosis and autophagy during the pathogenesis of Osteoarthritis within an experimental model. Apoptosis.

[10] Goldring M.B., Goldring S.R. (2010). Articular cartilage and subchondral bone in the pathogenesis of osteoarthritis. Ann. N. Y. Acad. Sci..

[11] Goldring M.B., Goldring S.R. (2007). Osteoarthritis. J. Cell. Physiol..

[12] Lane N.E., Brandt K., Hawker G., Peeva E., Schreyer E., Tsuji W., Hochberg M.C. (2011). OARSI-FDA initiative: Defining the disease state of osteoarthritis. Osteoarthr. Cartil..

[13] Tellegen A.R., Rudnik-Jansen I., Pouran B., de Visser H.M., Weinans H.H., Thomas R.E., Kik M.J.L., Grinwis G.C.M., Thies J.C., Woike N. (2018). Controlled release of celecoxib inhibits inflammation, bone cysts and osteophyte formation in a preclinical model of osteoarthritis. Drug Deliv..

[14] Loeser R.F., Goldring S.R., Scanzello C.R., Goldring M.B. (2012). Osteoarthritis: A disease of the joint as an organ. Arthritis Rheum..

[15] Awad H., Halawa F., Mostafa T., Atta H. (2006). Melatonin hormone profile in infertile males. Int. J. Androl..

[16] Rodríguez M.I., Escames G., López L.C., López A., García J.A., Ortiz F., Acuña-Castroviejo D. (2007). Chronic melatonin treatment reduces the age-dependent inflammatory process in senescence-accelerated mice. J. Pineal Res..

[17] Sugden D. (1983). Psychopharmacological effects of melatonin in mouse and rat. J. Pharmacol. Exp. Ther..

[18] Bilici D., Akpinar E., Kiziltunç A. (2002). Protective effect of melatonin in carrageenan-induced acute local inflammation. Pharmacol. Res..

[19] Carrillo-Vico A., Lardone P.J., Alvarez-Sánchez N., Rodríguez-Rodríguez A., Guerrero J.M. (2013). Melatonin: Buffering the Immune System. Int. J. Mol. Sci..

[20] Suzuki N., Somei M., Kitamura K., Reiter R.J., Hattori A. (2008). Novel bromomelatonin derivatives suppress osteoclastic activity and increase osteoblastic activity: Implications for the treatment of bone diseases. J. Pineal Res..

[21] Suzuki N., Hattori A. (2002). Melatonin suppresses osteoclastic and osteoblastic activities in the scales of goldfish. J. Pineal Res..

[22] Amstrup A.K., Sikjaer T., Heickendorff L., Mosekilde L., Rejnmark L. (2015). Melatonin improves bone mineral density at the femoral neck in postmenopausal women with osteopenia: A randomized controlled trial. J. Pineal Res..

[23] Maria S., Witt-Enderby P.A. (2014). Melatonin effects on bone: Potential use for the prevention and treatment for osteopenia, osteoporosis, and periodontal disease and for use in bone-grafting procedures. J. Pineal Res..

[24] Srinivasan V., Pandi-Perumal S.R., Spence D.W., Moscovitch A., Trakht I., Brown G.M., Cardinali D.P. (2010). Potential use of melatonergic drugs in analgesia: Mechanisms of action. Brain Res. Bull..

[25] Tan D.X., Manchester L.C., Sanchez-Barcelo E., Mediavilla M.D., Reiter R.J. (2010). Significance of high levels of endogenous melatonin in Mammalian cerebrospinal fluid and in the central nervous system. Curr. Neuropharmacol..

[26] Nakao N., Ono H., Yamamura T., Anraku T., Takagi T., Higashi K., Yasuo S., Katou Y., Kageyama S., Uno Y. (2008). Thyrotrophin in the pars tuberalis triggers photoperiodic response. Nature.

[27] Askari A., Ehrampoush E., Homayounfar R., Bahramali E., Farjam M. (2017). Serum insulin in pathogenesis and treatment of osteoarthritis. Med. Hypotheses.

[28] Maeda K., Kato Y., Ohgo S., Chihara K., Yoshimoto Y., Yamaguchi N., Kuromaru S., Imura H. (1975). Growth Hormone and Prolactin Release After Injection of Thyrotropin-Releasing Hormone in Patients with Depression. J. Clin. Endocrinol. Metab..

[29] Tashjian A.H., Barowsky N.J., Jensen D.K. (1971). Thyrotropin releasing hormone: Direct evidence for stimulation of prolactin production by pituitary cells in culture. Biochem. Biophys. Res. Commun..

[30] Abe E., Marians R.C., Yu W., Wu X.B., Ando T., Li Y., Iqbal J., Eldeiry L., Rajendren G., Blair H.C. (2003). TSH Is a Negative Regulator of Skeletal Remodeling. Cell.

[31] Sampath T.K., Simic P., Sendak R., Draca N., Bowe A.E., O’Brien S., Schiavi S.C., McPherson J.M., Vukicevic S. (2007). Thyroid-stimulating hormone restores bone volume, microarchitecture, and strength in aged ovariectomized rats. J. Bone Miner. Res..

[32] Kalev-Zylinska M.L., Hearn J.I., Rong J., Zhu M., Munro J., Cornish J., Dalbeth N., Poulsen R.C. (2018). Altered N-methyl D-aspartate receptor subunit expression causes changes to the circadian clock and cell phenotype in osteoarthritic chondrocytes. Osteoarthr. Cartil..

[33] Chan S., Debono M. (2010). Replication of cortisol circadian rhythm: New advances in hydrocortisone replacement therapy. Ther. Adv. Endocrinol. Metab..

[34] Kerrigan J.R., Veldhuis J.D., Leyo S.A., Iranmanesh A., Rogol A.D. (1993). Estimation of daily cortisol production and clearance rates in normal pubertal males by deconvolution analysis. J. Clin. Endocrinol. Metab..

[35] Linder B.L., Esteban N.V., Yergey A.L., Winterer J.C., Loriaux D.L., Cassorla F. (1990). Cortisol production rate in childhood and adolescence. J. Pediatr..

[36] Bierhaus A., Wolf J., Andrassy M., Rohleder N., Humpert P.M., Petrov D., Ferstl R., von Eynatten M., Wendt T., Rudofsky G. (2003). A mechanism converting psychosocial stress into mononuclear cell activation. Proc. Natl. Acad. Sci. USA.

[37] Naseem M., Parvez S. (2014). Role of melatonin in traumatic brain injury and spinal cord injury. Sci. World J..

[38] Lerner A.B., Case J.D., Takahashi Y., Lee T.H., Mori W. (1958). Isolation of melatonin, the pineal gland factor that lightens melanocytes. J. Am. Chem. Soc..

[39] Reiter R.J. (1998). Melatonin and human reproduction. Ann. Med..

[40] Stehle J.H., Saade A., Rawashdeh O., Ackermann K., Jilg A., Sebestény T., Maronde E. (2011). A survey of molecular details in the human pineal gland in the light of phylogeny, structure, function and chronobiological diseases. J. Pineal Res..

[41] Yonei Y., Hattori A., Tsutsui K., Okawa M., Ishizuka B. (2010). Effects of Melatonin: Basics Studies and Clinical Applications. Anti Aging Med..

[42] Magner J. (2014). Historical note: Many steps led to the “discovery” of thyroid-stimulating hormone. Eur. Thyroid J..

[43] Lechan R.M., Fekete C. (2006). The TRH neuron: A hypothalamic integrator of energy metabolism. Prog. Brain Res..

[44] Bianco A.C., Kim B.W. (2006). Deiodinases: Implications of the local control of thyroid hormone action. J. Clin. Investig..

[45] Russell W., Harrison R.F., Smith N., Darzy K., Shalet S., Weetman A.P., Ross R.J. (2008). Free triiodothyronine has a distinct circadian rhythm that is delayed but parallels thyrotropin levels. J. Clin. Endocrinol. Metab..

[46] Patel Y.C., Alford F.P., Burger H.G. (1972). The 24-hour plasma thyrotrophin profile. Clin. Sci..

[47] Lucke C., Hehrmann R., von Mayersbach K., von zur Mühlen A. (1977). Studies on circadian variations of plasma TSH, thyroxine and triiodothyronine in man. Acta Endocrinol..

[48] Lewis B.M., Dieguez C., Lewis M.D., Scanlon M.F. (1987). Dopamine stimulates release of thyrotrophin-releasing hormone from perfused intact rat hypothalamus via hypothalamic D2-receptors. J. Endocrinol..

[49] Oster H., Damerow S., Kiessling S., Jakubcakova V., Abraham D., Tian J., Hoffmann M.W., Eichele G. (2006). The circadian rhythm of glucocorticoids is regulated by a gating mechanism residing in the adrenal cortical clock. Cell Metab..

[50] Claustrat B., Brun J., Chazot G. (2005). The basic physiology and pathophysiology of melatonin. Sleep Med. Rev..

[51] Sae-Teaw M., Johns J., Johns N.P., Subongkot S. (2013). Serum melatonin levels and antioxidant capacities after consumption of pineapple, orange, or banana by healthy male volunteers. J. Pineal Res..

[52] Zhang H.M., Zhang Y. (2014). Melatonin: A well-documented antioxidant with conditional pro-oxidant actions. J. Pineal Res..

[53] Barsacchi R., Kusmic C., Damiani E., Carloni P., Greci L., Donato L. (1998). Vitamin E consumption induced by oxidative stress in red blood cells is enhanced by melatonin and reduced by N-acetylserotonin. Free Radic. Biol. Med..

[54] Banki K., Hutter E., Gonchoroff N.J., Perl A. (1999). Elevation of mitochondrial transmembrane potential and reactive oxygen intermediate levels are early events and occur independently from activation of caspases in Fas signaling. J. Immunol..

[55] Wölfler A., Caluba H.C., Abuja P.M., Dohr G., Schauenstein K., Liebmann P.M. (2001). Prooxidant activity of melatonin promotes fas-induced cell death in human leukemic Jurkat cells. FEBS Lett..

[56] Tan D.X., Manchester L.C., Terron M.P., Flores L.J., Reiter R.J. (2007). One molecule, many derivatives: A never-ending interaction of melatonin with reactive oxygen and nitrogen species?. J. Pineal Res..

[57] Melchiorri D., Reiter R.J., Sewerynek E., Hara M., Chen L., Nisticò G. (1996). Paraquat toxicity and oxidative damage. Reduction by melatonin. Biochem. Pharmacol..

[58] Tan D.X., Pöeggeler B., Reiter R.J., Chen L.D., Chen S., Manchester L.C., Barlow-Walden L.R. (1993). The pineal hormone melatonin inhibits DNA-adduct formation induced by the chemical carcinogen safrole in vivo. Cancer Lett..

[59] Reiter R.J., Tan D.X., Poeggeler B., Menendez-Pelaez A., Chen L.D., Saarela S. (1994). Melatonin as a free radical scavenger: Implications for aging and age-related diseases. Ann. N. Y. Acad. Sci..

[60] Reiter R.J., Melchiorri D., Sewerynek E., Poeggeler B., Barlow-Walden L., Chuang J., Ortiz G.G., Acuña-Castroviejo D. (1995). A review of the evidence supporting melatonin’s role as an antioxidant. J. Pineal Res..

[61] Osseni R.A., Rat P., Bogdan A., Warnet J.M., Touitou Y. (2000). Evidence of prooxidant and antioxidant action of melatonin on human liver cell line HepG2. Life Sci..

[62] Harms C., Lautenschlager M., Bergk A., Freyer D., Weih M., Dirnagl U., Weber J.R., Hörtnagl H. (2000). Melatonin is protective in necrotic but not in caspase-dependent, free radical-independent apoptotic neuronal cell death in primary neuronal cultures. FASEB J..

[63] Clapp-Lilly K.L., Smith M.A., Perry G., Harris P.L., Zhu X., Duffy L.K. (2001). Melatonin acts as antioxidant and pro-oxidant in an organotypic slice culture model of Alzheimer’s disease. Neuroreport.

[64] Hong Y., Kim H., Lee Y., Lee S., Kim K., Jin Y., Lee S.R., Chang K.T., Hong Y. (2014). Salutary effects of melatonin combined with treadmill exercise on cartilage damage. J. Pineal Res..

[65] Buyukavci M., Ozdemir O., Buck S., Stout M., Ravindranath Y., Savasan S. (2006). Melatonin cytotoxicity in human leukemia cells: Relation with its pro-oxidant effect. Fundam. Clin. Pharmacol..

[66] Keshavarzi S., Salehi M., Farifteh-Nobijari F., Hosseini T., Hosseini S., Ghazifard A., Ghaffari Novin M., Fallah-Omrani V., Nourozian M., Hosseini A. (2018). Melatonin modifies histone acetylation during in vitro maturation of mouse oocytes. Cell J..

[67] Albertini M.C., Radogna F., Accorsi A., Uguccioni F., Paternoster L., Cerella C., De Nicola M., D’Alessio M., Bergamaschi A., Magrini A. (2006). Intracellular pro-oxidant activity of melatonin deprives U937 cells of reduced glutathione without affecting glutathione peroxidase activity. Ann. N. Y. Acad. Sci..

[68] Cristofanon S., Uguccioni F., Cerella C., Radogna F., Dicato M., Ghibelli L., Diederich M. (2009). Intracellular prooxidant activity of melatonin induces a survival pathway involving NF-κB activation. Ann. N. Y. Acad. Sci..

[69] Gao C., Han H.B., Tian X.Z., Tan D.X., Wang L., Zhou G.B., Zhu S.E., Liu G.S. (2012). Melatonin promotes embryonic development and reduces reactive oxygen species in vitrified mouse 2-cell embryos. J. Pineal Res..

[70] Ali T., Rehman S.U., Shah F.A., Kim M.O. (2018). Acute dose of melatonin via Nrf2 dependently prevents acute ethanol-induced neurotoxicity in the developing rodent brain. J. Neuroinflamm..

[71] Kocyigit A., Guler E.M., Karatas E., Caglar H., Bulut H. (2018). Dose-dependent proliferative and cytotoxic effects of melatonin on human epidermoid carcinoma and normal skin fibroblast cells. Mutat. Res. Genet. Toxicol. Environ. Mutagen..

[72] Maharaj D.S., Anoopkumar-Dukie S., Glass B.D., Antunes E.M., Lack B., Walker R.B., Daya S. (2002). The identification of the UV degradants of melatonin and their ability to scavenge free radicals. J. Pineal Res..

[73] Ressmeyer A.R., Mayo J.C., Zelosko V., Sáinz R.M., Tan D.X., Poeggeler B., Antolín I., Zsizsik B.K., Reiter R.J., Hardeland R. (2003). Antioxidant properties of the melatonin metabolite N1-acetyl-5-methoxykynuramine (AMK): Scavenging of free radicals and prevention of protein destruction. Redox Rep..

[74] Tan D.X., Reiter R.J., Manchester L.C., Yan M.T., El-Sawi M., Sainz R.M., Mayo J.C., Kohen R., Allegra M., Hardeland R. (2002). Chemical and physical properties and potential mechanisms: Melatonin as a broad spectrum antioxidant and free radical scavenger. Curr. Top. Med. Chem..

[75] Ozturk G., Coşkun S., Erbaş D., Hasanoglu E. (2000). The effect of melatonin on liver superoxide dismutase activity, serum nitrate and thyroid hormone levels. Jpn. J. Physiol..

[76] Antolín I., Rodríguez C., Saínz R.M., Mayo J.C., Uría H., Kotler M.L., Rodríguez-Colunga M.J., Tolivia D., Menéndez-Peláez A. (1996). Neurohormone melatonin prevents cell damage: Effect on gene expression for antioxidant enzymes. FASEB J..

[77] Liu F., Ng T.B. (2000). Effect of pineal indoles on activities of the antioxidant defense enzymes superoxide dismutase, catalase, and glutathione reductase, and levels of reduced and oxidized glutathione in rat tissues. Biochem. Cell Biol..

[78] Shen Y.X., Xu S.Y., Wei W., Sun X.X., Liu L.H., Yang J., Dong C. (2002). The protective effects of melatonin from oxidative damage induced by amyloid beta-peptide 25-35 in middle-aged rats. J. Pineal Res..

[79] Shen Y.-X., Xu S.Y., Wei W., Sun X.X., Yang J., Liu L.H., Dong C. (2002). Melatonin reduces memory changes and neural oxidative damage in mice treated with D-galactose. J. Pineal Res..

[80] Wakatsuki A., Okatani Y., Shinohara K., Ikenoue N., Kaneda C., Fukaya T. (2001). Melatonin protects fetal rat brain against oxidative mitochondrial damage. J. Pineal Res..

[81] Benot S., Molinero P., Soutto M., Goberna R., Guerrero J.M. (1998). Circadian variations in the rat serum total antioxidant status: Correlation with melatonin levels. J. Pineal Res..

[82] Bonomini F., Favero G., Rodella L.F., Moghadasian M.H., Rezzani R. (2018). Melatonin modulation of Sirtuin-1 attenuates liver injury in a hypercholesterolemic mouse model. BioMed Res. Int..

[83] Wang Y.S., Li Y.Y., Cui W., Li L.B., Zhang Z.C., Tian B.P., Zhang G.S. (2017). Melatonin attenuates pain hypersensitivity and decreases astrocyte-mediated spinal neuroinflammation in a rat model of oxaliplatin-induced pain. Inflammation.

[84] Shao G., Tian Y., Wang H., Liu F., Xie G. (2015). Protective effects of melatonin on lipopolysaccharide-induced mastitis in mice. Int. Immunopharmacol..

[85] Kasahara T., Abe K., Mekada K., Yoshiki A., Kato T. (2010). Genetic variation of melatonin productivity in laboratory mice under domestication. Proc. Natl. Acad. Sci. USA.

[86] Semenova N.V., Madaeva I.M., Bairova T.A., Zhambalova R.M., Sholokhov L.F., Kolesnikova L.I. (2018). Association of the melatonin circadian rhythms with clock 3111T/C gene polymorphism in Caucasian and Asian menopausal women with insomnia. Chronobiol. Int..

[87] Pablos M.I., Reiter R.J., Ortiz G.G., Guerrero J.M., Agapito M.T., Chuang J.I., Sewerynek E. (1998). Rhythms of glutathione peroxidase and glutathione reductase in brain of chick and their inhibition by light. Neurochem. Int..

[88] Cuzzocrea S., Zingarelli B., Gilad E., Hake P., Salzman A.L., Szabó C. (1997). Protective effect of melatonin in carrageenan-induced models of local inflammation: Relationship to its inhibitory effect on nitric oxide production and its peroxynitrite scavenging activity. J. Pineal Res..

[89] Takaishi H., Kimura T., Dalal S., Okada Y., D’Armiento J. (2008). Joint diseases and matrix metalloproteinases: A role for MMP-13. Curr. Pharm. Biotechnol..

[90] Pei M., He F., Wei L., Rawson A. (2009). Melatonin enhances cartilage matrix synthesis by porcine articular chondrocytes. J. Pineal Res..

[91] Lim H.D., Kim Y.S., Ko S.H., Yoon I.J., Cho S.G., Chun Y.H., Choi B.J., Kim E.C. (2012). Cytoprotective and anti-inflammatory effects of melatonin in hydrogen peroxide-stimulated CHON-001 human chondrocyte cell line and rabbit model of osteoarthritis via the SIRT1 pathway. J. Pineal Res..

[92] Berenbaum F., Meng Q.J. (2016). The brain–joint axis in osteoarthritis: Nerves, circadian clocks and beyond. Nat. Rev. Rheumatol..

[93] Guo B., Yang N., Borysiewicz E., Dudek M., Williams J.L., Li J., Maywood E.S., Adamson A., Hastings M.H., Bateman J.F. (2015). Catabolic cytokines disrupt the circadian clock and the expression of clock-controlled genes in cartilage via an NFкB-dependent pathway. Osteoarthr. Cartil..

[94] Liu X., Xu Y., Chen S., Tan Z., Xiong K., Li Y., Ye Y., Luo Z.P., He F., Gong Y. (2014). Rescue of proinflammatory cytokine-inhibited chondrogenesis by the antiarthritic effect of melatonin in synovium mesenchymal stem cells via suppression of reactive oxygen species and matrix metalloproteinases. Free Radic. Biol. Med..

[95] Rong J., Zhu M., Munro J., Cornish J., McCarthy G.M., Dalbeth N., Poulsen R.C. (2019). Altered expression of the core circadian clock component PERIOD2 contributes to osteoarthritis-like changes in chondrocyte activity. Chronobiol. Int..

[96] Snelling S.J.B., Forster A., Mukherjee S., Price A.J., Poulsen R.C. (2016). The chondrocyte-intrinsic circadian clock is disrupted in human osteoarthritis. Chronobiol. Int..

[97] Yang W., Kang X., Liu J., Li H., Ma Z., Jin X., Qian Z., Xie T., Qin N., Feng D. (2016). Clock Gene Bmal1 Modulates Human Cartilage Gene Expression by Crosstalk with Sirt1. Endocrinology.

[98] Hong Y., Kim H., Lee S., Jin Y., Choi J., Lee S.-R., Chang K.-T., Hong Y. (2017). Role of melatonin combined with exercise as a switch-like regulator for circadian behavior in advanced osteoarthritic knee. Oncotarget.

[99] Lassová L., Niu Z., Golden E.B., Cohen A.J., Adams S.L. (2009). Thyroid hormone treatment of cultured chondrocytes mimics in vivo stimulation of collagen X mRNA by increasing BMP 4 expression. J. Cell. Physiol..

[100] Robson H., Siebler T., Stevens D.A., Shalet S.M., Williams G.R. (2000). Thyroid hormone acts directly on growth plate chondrocytes to promote hypertrophic differentiation and inhibit clonal expansion and cell proliferation. Endocrinology.

[101] Miura M., Tanaka K., Komatsu Y., Suda M., Yasoda A., Sakuma Y., Ozasa A., Nakao K. (2002). Thyroid hormones promote chondrocyte differentiation in mouse ATDC5 cells and stimulate endochondral ossification in fetal mouse tibias through iodothyronine deiodinases in the growth plate. J. Bone Miner. Res..

[102] Himeno M., Enomoto H., Liu W., Ishizeki K., Nomura S., Kitamura Y., Komori T. (2002). Impaired vascular invasion of Cbfa1-deficient cartilage engrafted in the spleen. J. Bone Miner. Res..

[103] Makihira S., Yan W., Murakami H., Furukawa M., Kawai T., Nikawa H., Yoshida E., Hamada T., Okada Y., Kato Y. (2003). Thyroid hormone enhances aggrecanase-2/ADAM-TS5 expression and proteoglycan degradation in growth plate cartilage. Endocrinology.

[104] Burch W.M., Lebovitz H.E. (1982). Triiodothyronine stimulates maturation of porcine growth-plate cartilage in vitro. J. Clin. Investig..

[105] Smith T.J., Murata Y., Horwitz A.L., Philipson L., Refetoff S. (1982). Regulation of glycosaminoglycan synthesis by thyroid hormone in vitro. J. Clin. Investig..

[106] Kato K., Otsuka T., Adachi S., Matsushima-Nishiwaki R., Natsume H., Kozawa O., Tokuda H. (2011). (-)-Epigallocatechin gallate inhibits thyroid hormone-stimulated osteocalcin synthesis in osteoblasts. Mol. Med. Rep..

[107] Kondo A., Otsuka T., Kato K., Matsushima-Nishiwaki R., Kuroyanagi G., Mizutani J., Tokuda H., Kozawa O. (2013). AMP-activated protein kinase regulates thyroid hormone-stimulated osteocalcin synthesis in osteoblasts. Int. J. Mol. Med..

[108] Endo T., Kobayashi T. (2013). Excess TSH causes abnormal skeletal development in young mice with hypothyroidism via suppressive effects on the growth plate. Am. J. Physiol. Endocrinol. Metab..

[109] Boutin A., Eliseeva E., Gershengorn M.C., Neumann S. (2014). β-Arrestin-1 mediates thyrotropin-enhanced osteoblast differentiation. FASEB J..

[110] Hase H., Ando T., Eldeiry L., Brebene A., Peng Y., Liu L., Amano H., Davies T.F., Sun L., Zaidi M. (2006). TNFalpha mediates the skeletal effects of thyroid-stimulating hormone. Proc. Natl. Acad. Sci. USA.

[111] Kaneki H., Guo R., Chen D., Yao Z., Schwarz E.M., Zhang Y.E., Boyce B.F., Xing L. (2006). Tumor Necrosis Factor Promotes Runx2 Degradation through Up-regulation of Smurf1 and Smurf2 in Osteoblasts. J. Biol. Chem..

[112] Steeve K.T., Marc P., Sandrine T., Dominique H., Yannick F. (2004). IL-6, RANKL, TNF-alpha/IL-1: Interrelations in bone resorption pathophysiology. Cytokine Growth Factor Rev..

[113] Sun L., Vukicevic S., Baliram R., Yang G., Sendak R., McPherson J., Zhu L.-L., Iqbal J., Latif R., Natrajan A. (2008). Intermittent recombinant TSH injections prevent ovariectomy-induced bone loss. Proc. Natl. Acd. Sci. USA.

[114] Hugues J.N., Reinberg A., Lagoguey M., Modigliani E., Sebaoun J. (1983). Biological rhythms of thyrotropin secretion. Ann. Med. Interne.

[115] Cooper M.S., Rabbitt E.H., Goddard P.E., Bartlett W.A., Hewison M., Stewart P.M. (2002). Osteoblastic 11β-Hydroxysteroid Dehydrogenase Type 1 Activity Increases with Age and Glucocorticoid Exposure. J. Bone. Miner. Res..

[116] Weinstein R.S., Wan C., Liu Q., Wang Y., Almeida M., O’Brien C.A., Thostenson J., Roberson P.K., Boskey A.L., Clemens T.L. (2010). Endogenous glucocorticoids decrease skeletal angiogenesis, vascularity, hydration, and strength in aged mice. Aging Cell.

[117] Tu J., Zhang P., Ji Z., Henneicke H., Li J., Kim S., Swarbrick M.M., Wu Y., Little C.B., Seibel M.J. (2019). Disruption of glucocorticoid signalling in osteoblasts attenuates age-related surgically induced osteoarthritis. Osteoarthr. Cartil..

[118] Debono M., Ghobadi C., Rostami-Hodjegan A., Huatan H., Campbell M.J., Newell-Price J., Darzy K., Merke D.P., Arlt W., Ross R.J. (2009). Modified-release hydrocortisone to provide circadian cortisol profiles. J. Clin. Endocrinol. Metab..

[119] Weitzman E.D., Fukushima D., Nogeire C., Roffwarg H., Gallagher T.F., Hellman L. (1971). Twenty-four hour pattern of the episodic secretion of cortisol in normal subjects. J. Clin. Endocrinol. Metab..

[120] Pace T.W., Mletzko T.C., Alagbe O., Musselman D.L., Nemeroff C.B., Miller A.H., Heim C.M. (2006). Increased stress-induced inflammatory responses in male patients with major depression and increased early life stress. Am. J. Psychiatry.

